# Chromosome level genome assembly of oriental armyworm *Mythimna separata*

**DOI:** 10.1038/s41597-023-02506-3

**Published:** 2023-09-08

**Authors:** Chao Xu, Jichao Ji, Xiangzhen Zhu, Ningbo Huangfu, Hui Xue, Li Wang, Kaixin Zhang, Dongyang Li, Lin Niu, Ran Chen, Xueke Gao, Junyu Luo, Jinjie Cui

**Affiliations:** 1 National Key Laboratory of Cotton Bio-breeding and Integrated Utilization, Institute of Cotton Research, Chinese Academy of Agricultural Sciences, Anyang, 455000 Henan China; 2 Hubei Insect Resources Utilization and Sustainable Pest Management Key Laboratory, College of Plant Science and Technology, Huazhong Agricultural University, Wuhan, 430070 Hubei China; 3 Zhengzhou Research Base, State Key Laboratory of Cotton Biology, School of Agricultural Sciences, Zhengzhou University, Zhengzhou, 450001 Henan China; 4 Western Agricultural Research Center, Chinese Academy of Agricultural Sciences, Changji, 831100 China; 5 College of Agronomy, Xinjiang Agricultural University, Urumqi, 830052 China

**Keywords:** Genome, Entomology

## Abstract

The oriental armyworm, *Mythimna separata*, is an extremely destructive polyphagous pest with a broad host range that seriously threatens the safety of agricultural production. Here, a high-quality chromosome-level genome was assembled using Illumina, PacBio HiFi long sequencing, and Hi-C scaffolding technologies. The genome size was 706.30 Mb with a contig N50 of 22.08 Mb, and 99.2% of the assembled sequences were anchored to 31 chromosomes. In addition, 20,375 protein-coding genes and 258.68 Mb transposable elements were identified. The chromosome-level genome assembly of *M. separata* provides a significant genetic resource for future studies of this insect and contributes to the development of management strategies.

## Background & Summary

**Fig. 1:**
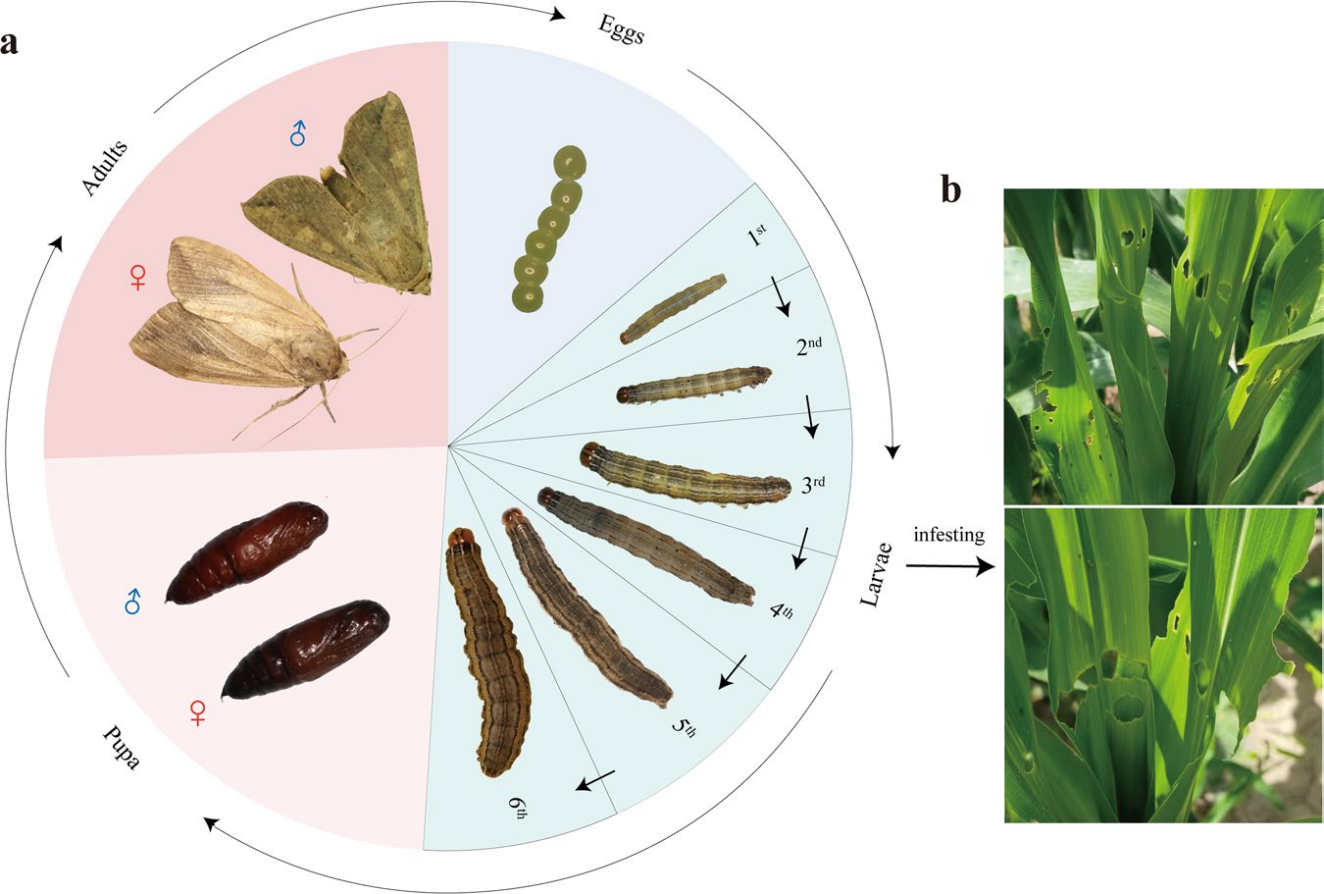
Development periods and damage of *M. separata*.

The oriental armyworm, *Mythimna separata* (Lepidoptera, Noctuidae), is a notorious polyphagous pest that is widely distributed in Asia, Australia, New Zealand, and several Pacific islands^1–3^ (Fig. 1a). This pest has a wide host range and poses a serious threat to the production of crops, particularly rice, maize, and wheat^4^ (Fig. 1b). The outbreak of *M. separata* in China from 2012 to 2013 threatened 1743.7 million hectares of farmland^5^, and this threat has continued in recent years^6–8^. This situation also occurs in other countries and regions where *M. separata* infestations are present^9^. In recent years, with the changes in global climate, crop planting structure, variety distribution, and cultivation system, *M. separata* has shown new characteristics in adaptability, breakout, and damage^10,11^. Due to its gregariousness, migration capability, polyphagy, and gluttony, *M. separata* was included in the list of first-class crop diseases and insect pests by the Chinese Ministry of Agriculture and Rural Affairs in 2020.

Previous studies have shown that polyphagous insects respond to toxic secondary metabolites produced by different host plants by inducing changes in the expression of genes related to detoxifying enzymes. Such changes may enhance the ability of polyphagous insects to adapt to host plants and develop resistance against pesticides^12^. However, the scarcity of genomic resources prevents the above hypothesis from being verified in *M. separata*. Although several *M. separata* genome assemblies were published in 2022 and 2023^13–16^, there are significant differences in the assembly method and quality of these genome assemblies. Hence, a high-quality chromosomal level genome is necessary to offer genetic resources and delve into the molecular mechanism of detoxification and host adaptation of *M. separata*, which will aid in providing theoretical support for optimizing management strategies for *M. separata*.

In the present study, we assembled a high-quality chromosome-level genome of *M. separata* by using a combination of Illumina short reads, PacBio high fidelity (HiFi) reads, and high-throughput chromosome conformation capture (Hi-C) data (Table 1). The genome assembly consisted of 172 contigs with a total length of 706.30 Mb, of which the contig N50 was 22.08 Mb. In addition, 99.2% of the draft assembly (700.63 Mb) was anchored to 31 chromosomes with a scaffold N50 of 23.00 Mb. We also identified 258.68 Mb of tandem repeats, accounting for 36.63% of the genome assembly. A total of 20,375 protein-coding genes were obtained, of which 98.53% were annotated. The results of phylogenetic analysis revealed that *M. separata* was diverged from *Helicoverpa armigera* approximately 25.91 Mya. Furthermore, 594 expanded gene families and 1329 contracted gene families were identified in *M. separata* genome. The high-quality chromosome-level genome assembly of *M. separata* will provide a genetic basis for further research on this polyphagous pest.

**Table 1:** Statistics of sequencing data of *M. separata* genome.

Library type	Insert Size (bp)	Raw Data (Gb)	Clean Data (Gb)	Coverage (X)
Illumina	350	59	58.72	88.62
PacBio	20000	986.03	70.62	99.98
Hi-C	350	—	76.08	114.81
RNA-Seq	150	13.94	13.66	19.5

## Methods

### Sample collection and genome sequencing

*M. separata* was collected from maize fields in Anyang City, Henan Province, China, and was subsequently reared in climate incubators at a temperature of 26 ± 1 °C with a relative humidity of 70% and a photoperiod of 14 h L:10 h D^17^. Genomic DNA was extracted from a single surface-sterilized male pupa using the QIAamp DNA Mini Kit (QIAGEN) for both Illumina and PacBio HiFi sequencing to prevent contamination from other individuals and microorganisms. For Hi-C sequencing, genomic DNA was extracted from a single male adult. Total RNA was extracted from adults using the TRIzol kit for transcriptome sequencing. The purity and integrity of genomic DNA and RNA were validated by the NanoDrop 2000C spectrophotometer (Thermo, Wilmington, DE, USA) and agarose gel electrophoresis (1.5%).

The paired-end libraries with a 350 bp inserted fragment were constructed and sequenced on the Illumina NovaSeq6000 platform following the manufacturer’s instruction. After removing adapter sequences and low-quality reads with HTQC (v1.92.310) software^18^, a total of 58.72 Gb clean reads were obtained for subsequent analyses. For PacBio HiFi sequencing, genomic DNA was sheared into ~15 Kb fragments using g-Tubes (Covaris, Woburn, MA, USA) and purified using 0.45 × AMPure PB beads (Beckman Coulter, Brea, CA, USA) for constructing SMRT bell libraries. Size selection was performed using the Sage ELF system (Sage Science, Beverly, MA) to collect SMRT bell libraries of 15–18 Kb. After annealing primers and binding Sequel II DNA polymerase to SMRT bell templates, sequencing was performed using 8 M SMRT cells on the Sequel II System (Biomarker Technologies Co., LTD, Beijing, China). A total of 986.03 Gb subreads were obtained and utilized to generate PacBio HiFi reads via the circular consensus sequencing (CCS) mode. Finally, a total of 70.62 Gb of CCS reads were produced, with an average read length of 16.67 kb, resulting in 99.98X coverages of the *M. separata* genome. The Hi-C library was constructed following the standard library preparation protocol^19^ and sequenced on the Illumina NovaSeq6000 platform, and 76.08 Gb of 150-bp paired-end clean reads were obtained.

### Genome survey and assembly

**Fig. 2:**
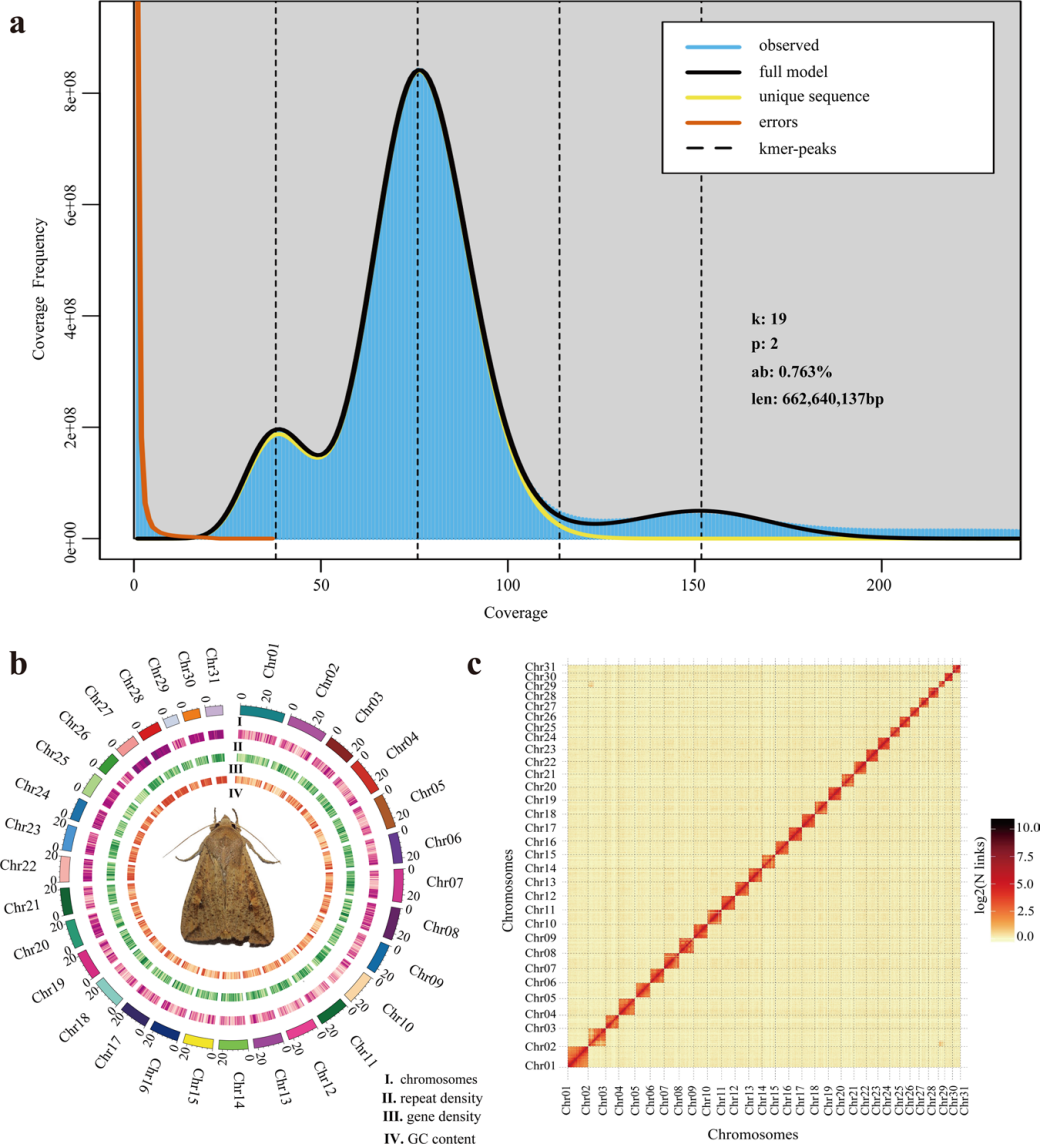
Genome assembly of *M. separata*. (**a**) Genome scope profiles of 19-mer analysis. (**b**) Circle genome landscape of *M. separata*. Circle I represents chromosomes, while circles II-IV indicate repeat density, gene density, and GC content of each respective chromosome. (**c**) Hi-C interactive heatmap of *M. separata*. Color indicates the intensity of the interaction signal. The darker the color, the higher the intensity.

**Fig. 3:**
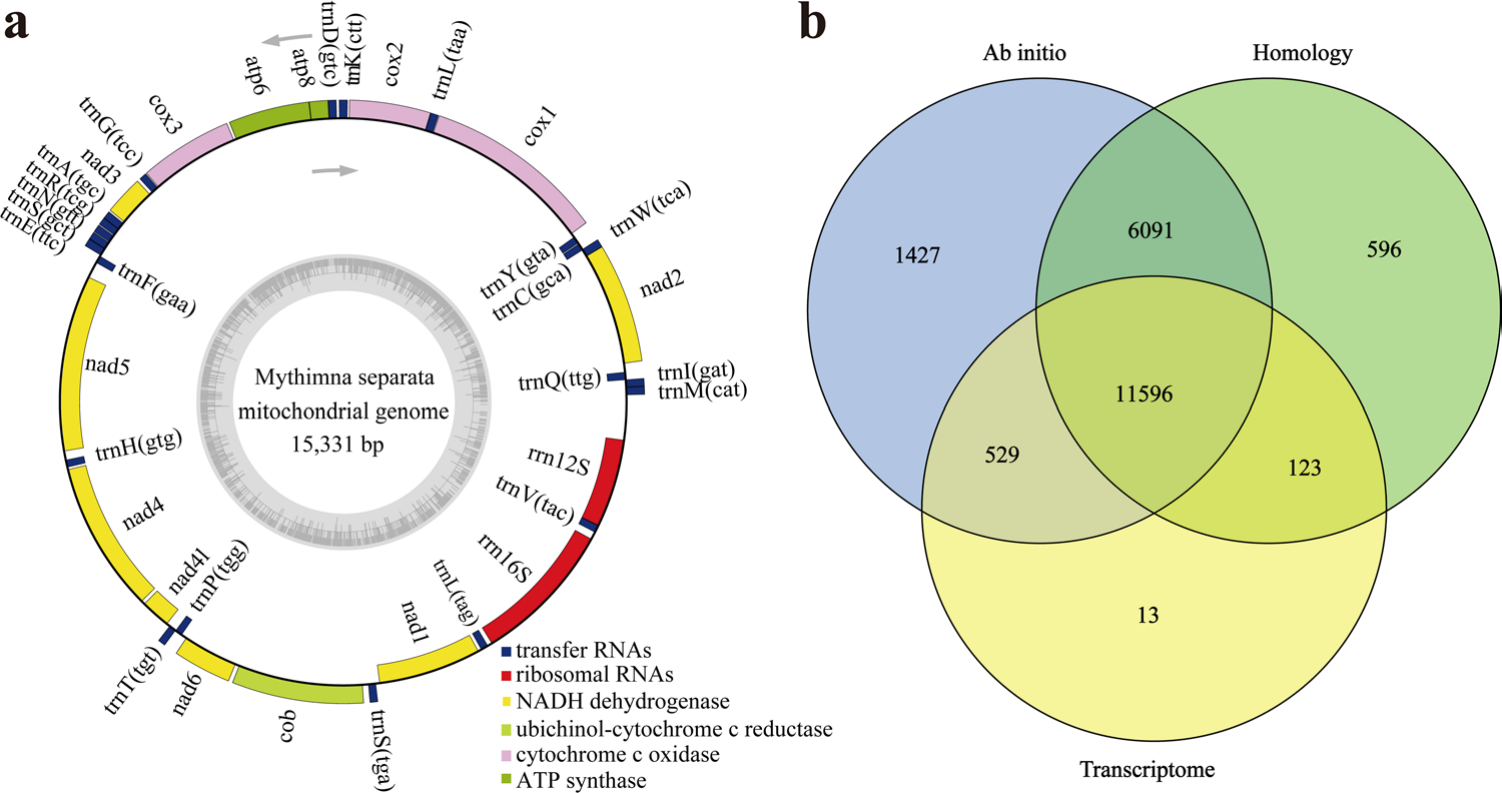
Mitochondrial genome assembly and protein-coding gene prediction of *M. separata*. (**a**) Circular map of *M. separata* mitochondrial genome. Gene map presents 37 annotated genes of different functional groups. (**b**) Venn diagrams of protein-coding genes obtained from three prediction methods.

Genome survey was essential to estimate the main characteristics, including genome size, repetitive sequence content and heterozygosity. The k-mer (K = 19) frequencies were constructed based on Illumina clean short-reads using Jellyfish (v2.2.10)^20^ and used to perform genome survey by GenomeScope (v2.0)^21^. The estimated genome scale of *M. separata* was 662.64 Mb, with a repetitive content of 39.00% and a heterozygosity of 0.76% (Fig. 2a). Subsequently, CCS reads were submitted to Hifiasm (v0.15.1)^22^ and assembled with default parameters. After filtering haplotypic duplicates using purge_dups^23^ with parameters of ‘−2 -T cutoffs -c PB.base.cov’, the *M. separata* genome assembly was generated. The assembly consisted of 172 contigs with a total length of 706.30 Mb and a contig N50 of 22.08 Mb. The clean Hi-C reads were aligned to the draft genome assembly using BWA (0.7.10)^24^ with default parameters. The uniquely aligned read pairs were further processed using HiC-Pro (v2.10.0)^25^ to assess and eliminate the invalid read pairs, including dangling-end, re-ligation, self-cycle, and dumped pairs. A total of 88,824,108 valid interaction pairs for scaffold correction were used to cluster, order, and orient contigs onto chromosomes using LACHESIS (v2e27abb)^26^ with default parameters. Finally, 147 scaffolds were anchored to 31 chromosomes with a scaffold N50 of 23.00 Mb, covering a span of 700.63 Mb and representing 99.2% of the draft genome assembly (Fig. 2b,c, Table 2). In addition, the mitochondrial genome of *M. separata* was assembled through mitoZ^27^ and NOVOplasty^28^, and subsequently annotated using MITOS^29^ and GeSeq^30^ (Fig. 3a, Table 3).

**Table 2:** Statistics of Hi-C assembly results.

Pseudomolecule	No. Cluster	Cluster Length (bp)	No. Order	Order Length (bp)
Chr1	1	34,940,390	1	34,940,390
Chr2	6	32,516,418	4	30,570,595
Chr3	4	31,168,564	1	22,398,001
Chr4	5	27,889,153	3	27,775,276
Chr5	5	28,330,987	4	26,740,777
Chr 6	6	26,064,612	4	23,807,975
Chr7	4	26,030,776	3	25,981,772
Chr8	8	25,415,376	7	25,213,998
Chr9	4	24,633,254	2	23,716,560
Chr10	9	24,135,932	9	24,135,932
Chr11	5	23,984,291	3	23,474,426
Chr12	2	23,502,669	2	23,502,669
Chr13	3	23,494,354	1	22,998,590
Chr14	2	22,982,399	2	22,982,399
Chr15	3	23,242,639	3	23,242,639
Chr16	3	22,701,724	3	22,701,724
Chr17	3	22,353,031	2	22,317,867
Chr18	7	23,439,917	7	23,439,917
Chr19	1	22,078,253	1	22,078,253
Chr20	5	22,346,897	4	22,005,563
Chr21	2	21,224,985	2	21,224,985
Chr22	2	20,477,231	1	20,180,707
Chr23	1	20,153,354	1	20,153,354
Chr24	11	17,358,959	11	17,358,959
Chr25	1	17,764,251	1	17,764,251
Chr26	3	17,426,045	2	15,738,203
Chr27	6	17,560,105	3	16,490,836
Chr28	5	16,964,831	5	16,964,831
Chr29	6	13,194,283	4	11,046,490
Chr30	3	13,680,854	2	13,427,725
Chr31	6	13,569,241	6	13,569,241
**Total** (**Ratio %**)	132 (60)	70,0625,775 (99.2)	104 (78.79)	677,944,905 (96.76)

**Table 3:** Annotation of *M. separata* mitochondrial genome.

Gene Name	Start Position	End Position	Length (bp)	Direction
trnM(cat)	57	124	68	forward
trnI(gat)	125	189	65	forward
trnQ(ttg)	187	255	69	reverse
nad2	330	1316	987	forward
trnW(tca)	1315	1384	70	forward
trnC(gca)	1377	1441	65	reverse
trnY(gta)	1447	1511	65	reverse
cox1	1547	3044	1498	forward
trnL2(taa)	3048	3114	67	forward
cox2	3115	3783	669	forward
trnK(ctt)	3797	3867	71	forward
trnD(gtc)	3888	3954	67	forward
atp8	3955	4110	156	forward
atp6	4110	4778	669	forward
cox3	4799	5569	771	forward
trnG(tcc)	5575	5639	65	forward
nad3	5664	5990	327	forward
trnA(tgc)	6003	6069	67	forward
trnR(tcg)	6069	6133	65	forward
trnN(gtt)	6139	6205	67	forward
trnS1(gct)	6209	6274	66	forward
trnE(ttc)	6275	6341	67	forward
trnF(gaa)	6350	6416	67	reverse
nad5	6568	8082	1515	reverse
trnH(gtg)	8167	8232	66	reverse
nad4	8252	9568	1317	reverse
nad4l	9599	9859	261	reverse
trnT(tgt)	9889	9953	65	forward
trnP(tgg)	9954	10018	65	reverse
nad6	10065	10547	483	forward
cob	10580	11665	1086	forward
trnS2(tga)	11714	11780	67	forward
nad1	11812	12711	900	reverse
trnL1(tag)	12739	12806	68	reverse
rrn16S	12844	14101	1258	reverse
trnV(tac)	14166	14231	66	reverse
rrn12S	14232	15003	772	reverse

### Genomic repeat annotation

Repeat sequences mainly include tandem repeats and interspersed repeats, with the latter mainly being transposable elements (TE). The repeat sequences of TE were annotated using a combination of homology-based and *de novo* approaches. We initially customized a *de novo* repeat library using RepeatModeler^31^ and LTR_retriever (v2.8)^32^ based on the assembly sequences with default parameters. The predicted repeats were subsequently classified using PASTEClassifier (v1.0)^33^, and the results were combined with databases of Repbase^34^, REXdb (v3.0)^35^, and Dfam (v3.2)^36^ to construct a species-specific TE library without redundancy. TE sequences were identified by homology search against the library using RepeatMasker (v4.10)^37^. A total of 258.68 Mb TE sequences were obtained, accounting for 36.63% genome assembly. In addition, 23.64 Mb (3.35%) tandem repeats were identified using MISA (v2.1)^38^ and NCRF^39^ (Table 4).

**Table 4:** Statistics of repeat elements of *M. separata* genome.

Repeat types	Number	Length (bp)	Percent (%)
SINE	55307	8037713	1.14
LTR	724260	120106832	17
LINE	334544	66072296	9.35
DIRS	565	111357	0.02
DNA transposons	321395	64356610	9.11
Tandem repeats	217211	23635406	3.35
Total	1653282	282320232	39.98

### Gene prediction and functional annotation

Three approaches, including *de novo* prediction, homolog-based and transcriptome-based methods, were combined to perform gene prediction after eliminating the interference of repeat sequences in the *M. separata* genome. The *de novo* gene models were predicted using two ab initio gene-prediction software tools of Augustus (v2.4)^40^ and SNAP^41^ with default parameters. Homology-based gene prediction was conducted using GeMoMa (v1.7)^42^ against the protein sequences of lepidopteran insects *B. mori, H. armigera, S. frugiparda*, and *S. litura* downloaded from GenBank. For transcriptome-based gene prediction, the RNA-seq reads were assembled into unigenes using Trinity (v2.11)^43^, and resulting unigenes were then used to identify protein-coding genes via PASA (v2.0.2)^44^. Finally, gene models obtained from these three methods were integrated into a unified gene set using EVidenceModeler (v1.1.1)^45^ with default parameters. As a result, 20,375 protein-coding genes were identified from *M. separata* genome (Fig. 3b).

**Fig. 4:**
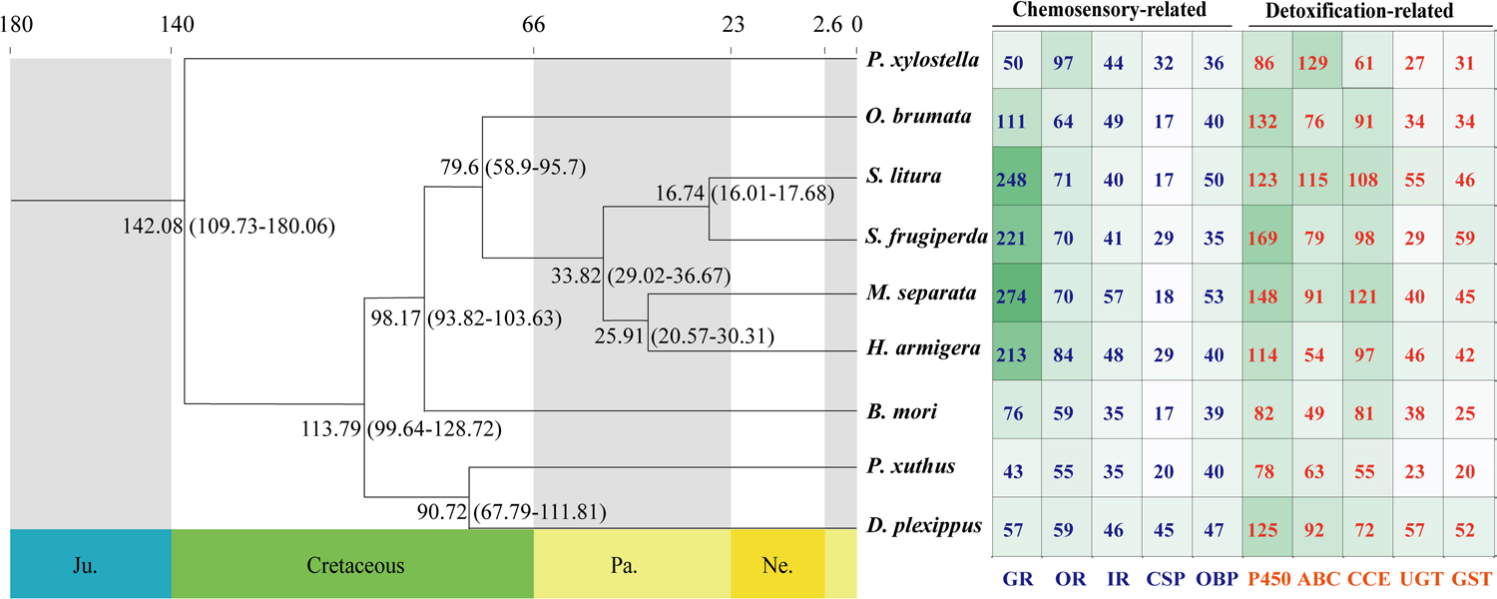
Divergence time and distribution of detoxification and chemosensory genes in *M. spearata* and other eight lepidopteran insects. The branch node values indicate the inferred divergence time between species. The numbers in the right cells indicate the scale of the corresponding gene family in each species. The darker the background color of cells, the more the genes encoded in the corresponding species.

In order to perform functional annotation of the protein-coding genes, we aligned predicted genes against databases including NR, GO, KEGG, EggNOG, KOG, TrEMBL, InterPro and Swiss-Prot using BLAST (v2.2.31) with a threshold of 1e^−5^. Finally, 98.53% (20075/20375) of protein-coding genes were annotated (Table 5). The detoxification-related genes cytochrome P450 (P450), ATP-binding cassette (ABC), Carboxyl/cholinesterase (CCE), UDP-glycosyltransferases (UGTs), and glutathione-S-transferase (GST), as well as the chemosensory-related genes of ionotropic receptors (IRs), chemosensory proteins (CSPs), and odorant binding proteins (OBPs), were further annotated using BLASTP (E < 10^−5^). To annotate genes associated with gustatory receptors (GRs) and odorant receptors (ORs), we identified candidate loci through TBLASTN with E-values < 10^−5^ and predicted gene structures using GeneWise (v2.2.0)^46^ (Fig. 4).

**Table 5:** Statistics of functional annotation in *M. separata* genome.

Annotation type	Number	Percent (%)
GO	14397	70.66
KEGG	15587	76.5
KOG	11244	55.19
Pfam	15558	76.36
Swissprot	13447	66
TrEMBL	19987	98.1
eggNOG	14333	70.35
NR	20034	98.33
Total annotated genes	20075	98.53
Predicted protein-coding genes	20375	—

### Phylogenetic analysis

The protein sequences of seventeen insects, including eight Lepidoptera insects and nine others associated with Diptera, Coleoptera, Hymenoptera, Hemiptera, and Odonata, were downloaded from NCBI for phylogenetic analysis (Table 6). The orthologous gene families were detected using OrthoFinder (v2.4.0)^47^ and annotated based on the PANTHER^48^ database. The single-copy orthologous genes were aligned using MAFFT (v7.205)^49^, and ambiguously aligned regions were removed by applying Gblocks (v0.91b)^50^ with default parameters. The phylogenetic trees were constructed by IQ-TREE (v1.6.10)^51^ with 1000 bootstrap replicates and the best model of LG + F + I + G4. The divergence time between different species was estimated using MCMCtree (PAML^52^ package) based on the fossil records acquired from TimeTree database (http://www.timetree.org/). Furthermore, the results obtained from phylogenetic trees, which included divergence time, were employed to identify the expansion and contraction of gene families using CAFE (v5.0)^53^ with a p-value threshold ≤ 0.05.

**Table 6:** Download link of 17 insect genomes used for phylogenetic analysis.

Species	Download link
*C. septempunctata*	https://www.ncbi.nlm.nih.gov/genome/72445?genome_assembly_id=1620206
*S. litura*	ftp://ftp.ncbi.nlm.nih.gov/genomes/all/GCF/002/706/865/GCF_002706865.1_ASM270686v1
*N. lugens*	https://www.ncbi.nlm.nih.gov/genome/2941?genome_assembly_id=986525
*A. arabiensis*	https://www.ncbi.nlm.nih.gov/genome/11544?genome_assembly_id=1582864
*O. brumata*	https://www.ncbi.nlm.nih.gov/genome/39883?genome_assembly_id=245790
*S. frugiperda*	https://www.ncbi.nlm.nih.gov/genome/10985?genome_assembly_id=1839534
*P. xuthus*	https://www.ncbi.nlm.nih.gov/genome/13942?genome_assembly_id=219896
*L. heterotoma*	https://www.ncbi.nlm.nih.gov/genome/17698?genome_assembly_id=1491693
*B. mori*	https://www.ncbi.nlm.nih.gov/genome/76?genome_assembly_id=1491718
*H. armigera*	https://www.ncbi.nlm.nih.gov/genome/13316?genome_assembly_id=1866364
*P. xylostella*	https://www.ncbi.nlm.nih.gov/genome/11570?genome_assembly_id=1806547
*A. mellifera*	https://www.ncbi.nlm.nih.gov/genome/48?genome_assembly_id=403979
*I. elegans*	https://www.ncbi.nlm.nih.gov/genome/50386?genome_assembly_id=1749491
*D. melanogaster*	https://www.ncbi.nlm.nih.gov/genome/?term=Drosophila+melanogaster
*D. plexippus*	https://www.ncbi.nlm.nih.gov/genome/11702?genome_assembly_id=748550
*P. pyralis*	https://www.ncbi.nlm.nih.gov/genome/?term=Photinus +pyralis
*A. gossypii*	https://www.ncbi.nlm.nih.gov/genome/17818?genome_assembly_id=1910936

### Genome synteny analysis

**Fig. 5:**
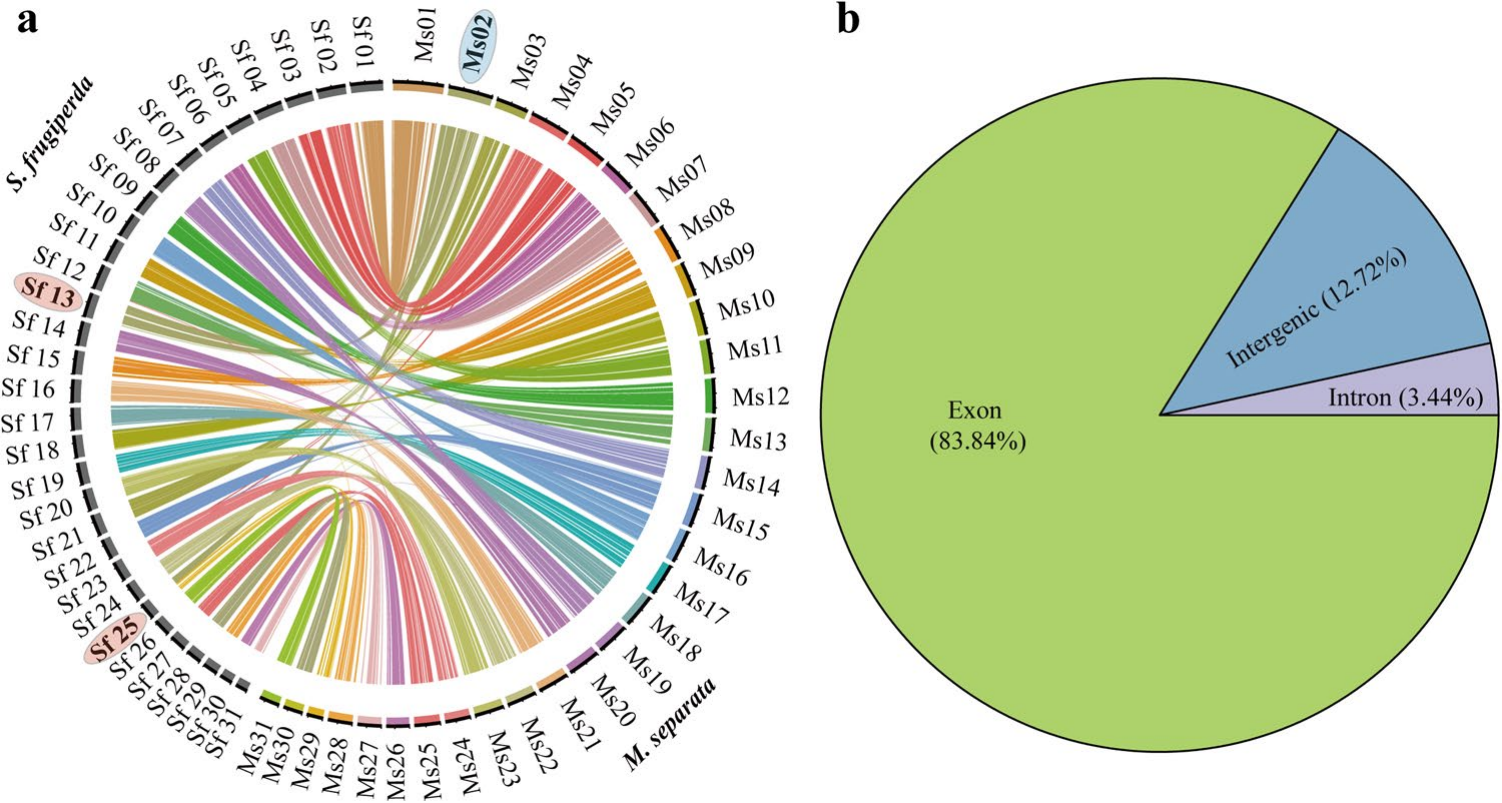
Genome synteny and verification of protein-coding genes of *M. separata* genome. (**a**) Whole-genome synteny between *M. spearata* and *Spodoptera frugiperda*. (**b**) RNA-seq clean data verified the accuracy of protein-coding gene prediction.

In order to perform genome synteny analysis of *M. separata* with *Spodoptera frugiperda*, the similar gene pairs were identified using Diamond (v0.9.29)^54^ with default parameters. All genes in synteny blocks were obtained by MCScanX^55^, and synteny blocks were then visualized across chromosomes using CIRCOS (v 0.69–9)^56^. Only one fission event was identified between *M. separata* and *Spodoptera frugiperda*, which suggested a high degree of concordance between them (Fig. 5a).

## Data Records

The raw data of Illumina sequencing, PacBio HiFi sequencing and Hi-C sequencing of the *Mythimna separata* genome was deposited at the NCBI SRA database with the accession number of SRP433040^57^. The final assembled *Mythimna separata* genome has been submitted to NCBI under accession number GCA_030763345.1^58^. The annotation files of the *Mythimna separata* genome have been deposited at figshare^59^.

## Technical Validation

### Evaluation of the genome assembly

The integrity and accuracy of genome assembly were verified from three aspects: Firstly, the clean reads acquired from Illumina sequencing were aligned against the genome assembly using BWA^24^. The results revealed that 99.26% of Illumina reads were aligned to the genome assembly. Secondly, the Core Eukaryotic Genes Mapping Approach (CEGMA) database contained 458 conserved core eukaryotic genes, of which 431 (94.10%) were identified in *M. separata* genome. Finally, the completeness of the genome assembly was evaluated using BUSCO (v4)^20^ with parameters of ‘-m prot -f -l eukaryota_odb9’, and 98.74% of the conserved core BUSCOs were identified in the genome of *M. separata*. These results showed that we obtained the high-quality *M. separata* genome assembly. Meanwhile, the contig N50 in our assembly was 22.08 Mb, which was significantly higher than the 7.31 Mb in recent assembly version of *M. separata*^13^. The scaffold N50 in our assembly was improved to 23.00 Mb, which was slightly higher than the 22.68 Mb in other recent assembly version of *M. separata*^15^ (Table 7).

**Table 7:** Comparative statistic of five *M. separata* genome assemblies.

Genome assembly	This study	Jiang *et al*.^13^	Kakeru *et al*.^14^	Zhao *et al*.^15^	CAU (2021)^16^
Genome size (Mb)	706.30	665.7	682	688.38	700.25
Assembly level	Chromosome	Chromosome	Contig	Chromosome	Chromosome
Number chromosomes	31	31	—	31	31
Contig N50 (Mb)	22.08	7.31	2.7	22.58	3.4
Scaffold N50 (Mb)	23.00	22.2	—	22.68	23.00
BUSCO complete rate of the genome	98.74%	98%	99.2%	98.2%	—
GC content (%)	38.70%	38.5%	38.6%	—	—
Number of genes	20375	17067	21970	17549	—
Repeat (%)	39.98%	47.1%	46.59%	45%	—

To assess the quality of chromosome assembly, the assembly was sheared into 100 kb bins, and the intensity of the interaction pairs was used to plot heatmaps. The Hi-C heatmap showed that the intensity of interaction along the diagonals was obviously higher than that at non-diagonal positions in 31 distinct chromosomes.

### Evaluation of gene prediction

The BUSCO analysis was also used to assess the results of gene prediction. The 98.74% (942/954) of the BUSCOs were identified from the predicted gene set of our genome, which was slightly higher than the 98% and 98.2% in other recent *M. separata* assembly version^13,15^. Meanwhile, 83.84% of the RNA-seq data were aligned to the predicted exons (Fig. 5b). These results confirmed the completeness and accuracy of gene prediction across *M. separata* genome. In addition, 20,375 protein-coding genes were identified in our genome assembly, which was significantly more than the 17549 protein-coding genes in the best available recent reference genome assembly version of *M. separata*^15^. We further compared the set of protein-coding genes in the two genome assemblies using local BLASTN with E-values < 10^−5^. A total of 16,398 protein-coding genes were identified in both two genome assemblies, and 2,828 protein-coding genes were identified only in our genome assembly.

### Comparative genomic analysis

**Fig. 6:**
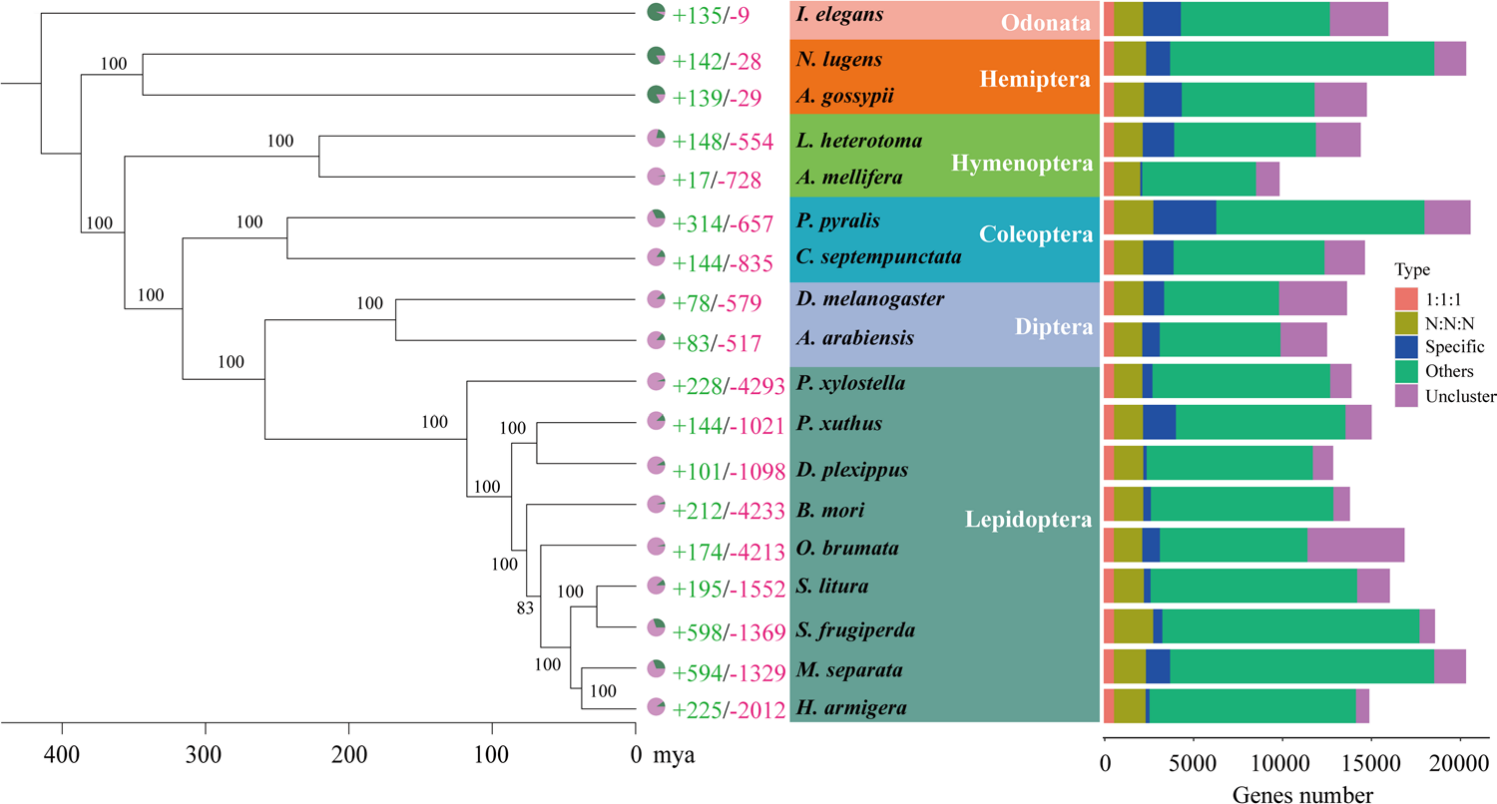
Phylogenetic tree of *M. spearata* together with 17 other insects. The maximum likelihood phylogenomic tree was calculated based on 565 single-copy genes. The numbers of expanded gene families (green) and contracted gene families (red) are displayed to the right of each species branch. The coloured histogram indicates that genes of each species were categorized into five groups: 1:1:1 (single copy orthologous genes in common gene families); N: N: N (multiple copy orthologous genes in common gene common gene families); Specific (genes from unique gene families from each species); Other (genes that do not belong to any of the above ortholog categories); Unclustered (genes which are not clustered into any family).

**Fig. 7:**
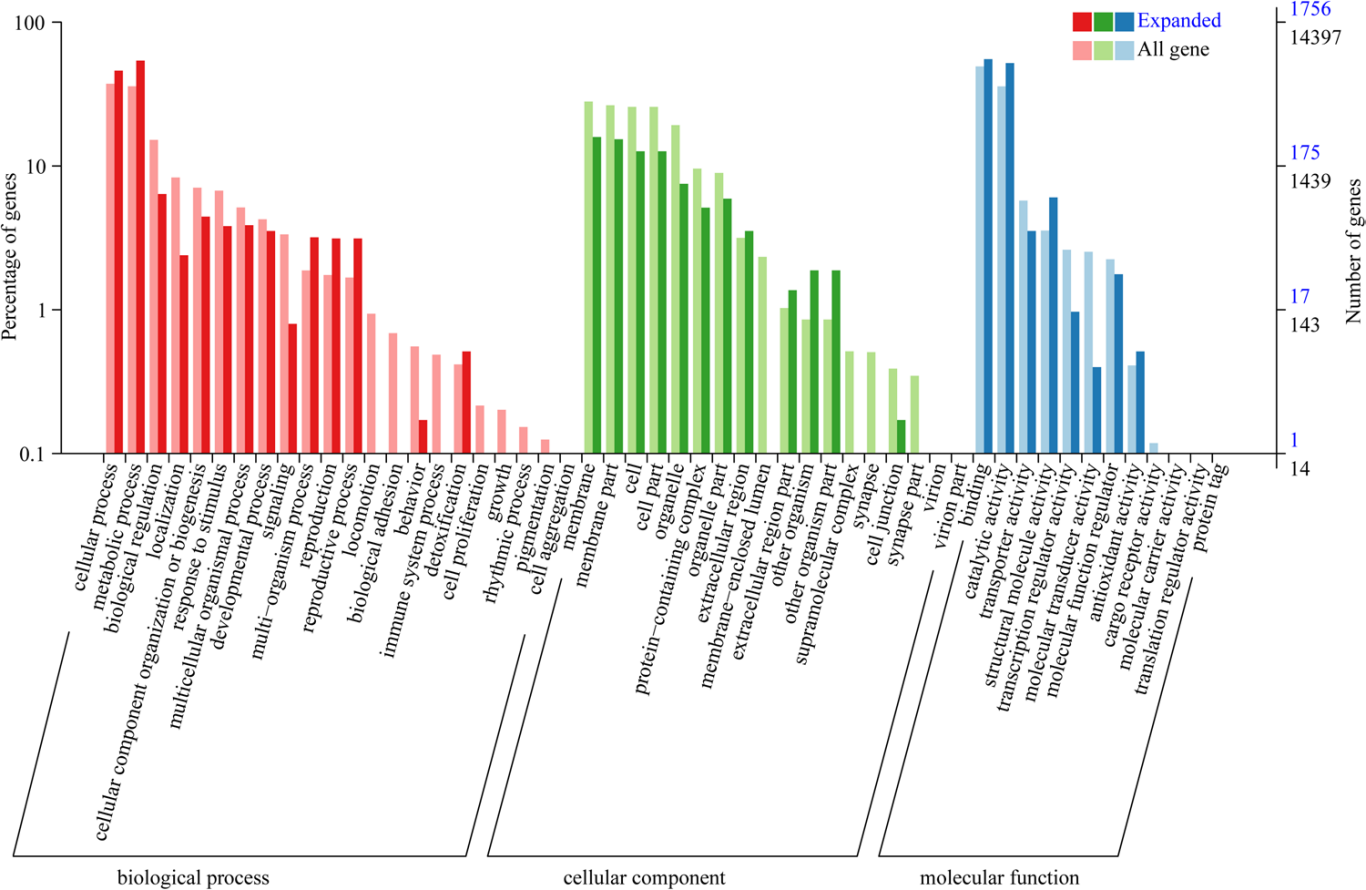
Go enrichment analyses of *M. separata* expanded gene families.

**Fig. 8:**
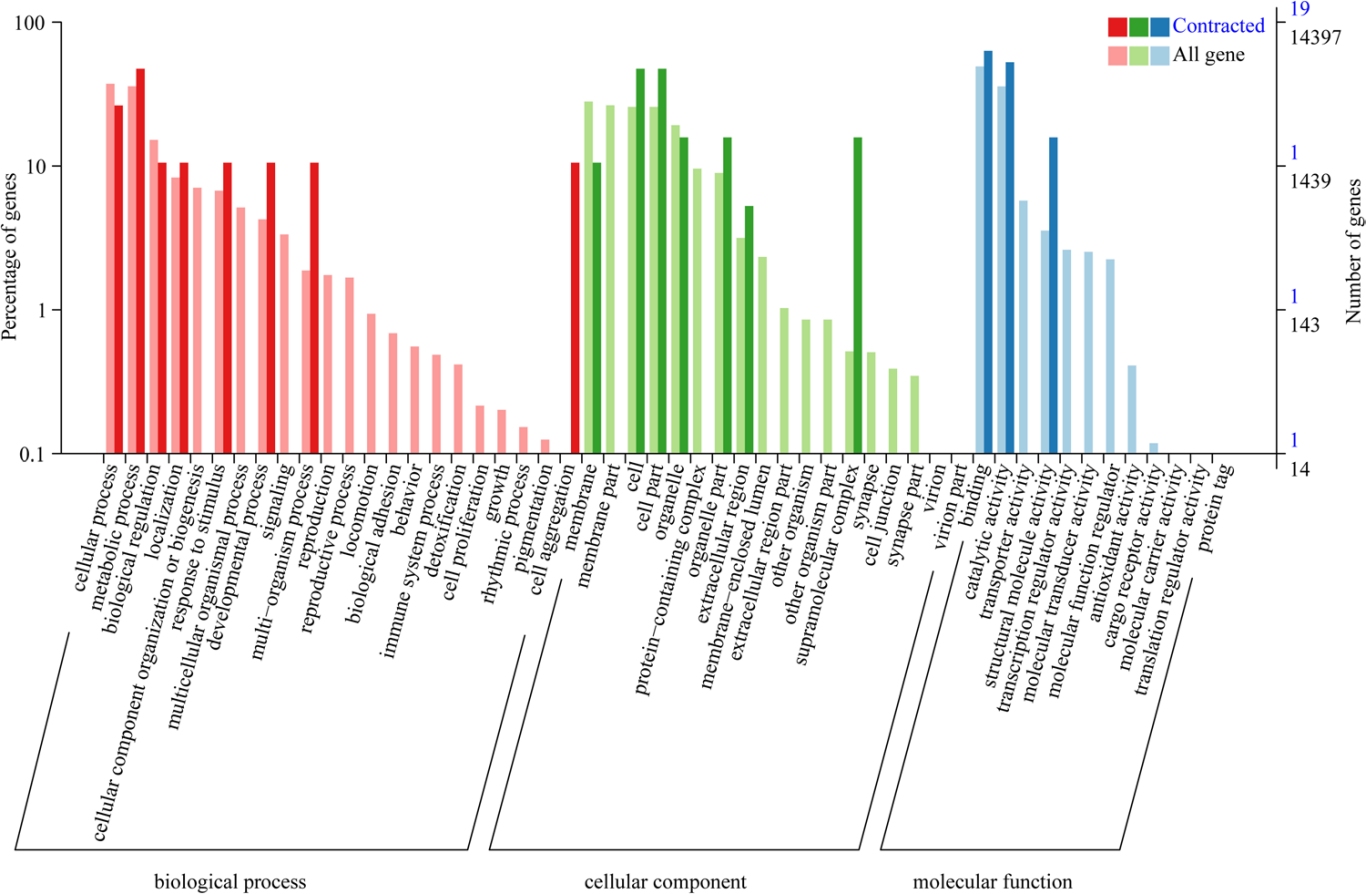
Go enrichment analyses of *M. separata* contracted gene families.

A total of 27,002 orthologous gene families were identified from the 18 insect species, of which 565 single-copy orthologous gene families were used for phylogenetic analysis (Table S1). The results of phylogenetic analysis indicated that Lepidoptera insects speciated from their common ancestor later than Diptera, Coleoptera, Hymenoptera and Hemiptera (Fig. 6). *M. separata* and *H. armigera* were found to cluster into a single clade within Lepidoptera and to diverge at approximately 25.29 (20.57–30.31) million years ago (mya). In addition, *M. separata* and *S. frugiperda* were estimated to diverge at approximately 33.82 (29.02–36.67) Mya ago. Meanwhile, GO enrichment analysis revealed that the 594 expanded gene families in *M. separata* genome mainly involved “DNA integration” (GO:0015074), “nucleosome” (GO: 0000786), and “RNA-directed DNA polymerase activity” (GO:0003964), while the 1329 contracted gene families mainly involved “regulation of signal transduction” (GO:0009966), “membrane” (GO:0016020), and “serine-type endopeptidase activity” (GO:0004252) (Figs. 7, 8, Tables S2, S3).

### Supplementary information

Supplementary tables

## Data Availability

All bioinformatic tools and softwares for data analysis in this study were used according to the manuals, and the version and code/parameters of software have been introduced in Methods section. No custom code was used.
